# Spanish Adaptation of the Experiential Approach Scale (EAS)

**DOI:** 10.3390/ijerph182412873

**Published:** 2021-12-07

**Authors:** Salvador Reyes-Martín, Mónica Hernández-López, Miguel Rodríguez-Valverde

**Affiliations:** Department of Psychology, Campus las Lagunillas s/n, University of Jaén, 23071 Jaén, Spain; srm00046@red.ujaen.es (S.R.-M.); mhlopez@ujaen.es (M.H.-L.)

**Keywords:** psychological inflexibility, experiential avoidance, experiential approach, acceptance and commitment therapy, EAS

## Abstract

Psychological inflexibility is a transdiagnostic dimension associated to psychological distress and poor mental health and quality of life. While multiple instruments have been developed for the assessment of patterns of inflexible responding to aversive private events (e.g., unwanted cognitions and emotions), the Experiential Approach Scale (EAS) is the first instrument specifically designed to assess inflexible responding to appetitive private events (e.g., desired affective states). In this study, we explored the factor structure, internal consistency, and convergent validity of a Spanish adaptation of the EAS with a convenience sample of college students from Spain (*n* = 206; 79% female). A two-factor solution demonstrated very good fit to the data and was similar to the original two-subscale EAS structure: Anxious Clinging and Experience Prolonging. The scale showed adequate overall (α = 0.85) and subscale (αs: 0.90 and 0.89) internal consistency. Unlike the original instrument, both subscales were uncorrelated. Anxious Clinging correlated positively with experiential avoidance and with measures of negative affect and psychopathology, and negatively with positive affect, subjective happiness, and life satisfaction. In turn, Experience Prolonging correlated negatively with psychopathology and positively with positive affect, subjective happiness, and life satisfaction. Our results point to Anxious Clinging as the only EAS subscale contributing to psychological inflexibility.

## 1. Introduction

In the last 25 years, we have witnessed the rise of a new generation of behavioral therapies, the so-called Third Wave therapies [1]. This term has been used to designate a diverse group of therapies that focus on how individuals respond to their own behavior, and specifically to aversive private events (e.g., negative thoughts, fears, distressing memories). These therapies share the assumption that deliberate attempts to control and avoid aversive private events might exacerbate their very frequency and intensity, leading to psychological suffering and psychopathology [2,3]. A key element to these therapies is the idea that open awareness and acceptance of private events are the most useful ways of promoting meaningful positive changes in people’s lives.

### 1.1. Experiential Avoidance: A Key Component of Psychological Inflexibility

Acceptance and Commitment Therapy (ACT) [4,5,6] is one of the most representative and empirically supported of this Third Wave [7]. The ACT model of psychopathology assumes that most psychological disorders result from psychological inflexibility, characterized by cognitive fusion (the dominance of the verbal functions of private events over actual environmental contingencies) that leads to rigid patterns of experiential avoidance (persistent avoidance of unwanted cognitions and emotions and the situational triggers that occasion them) [2,3]. Experiential avoidance may provide relief in the short run, but it will frequently interfere with engagement in valued actions, bringing about more distress. This, in turn, could trigger more avoidance, in a sort of vicious cycle. There is broad evidence that experiential avoidance and, more generally, the lack of flexibility regarding private events is associated with emotional distress and poor mental health and quality of life [8,9,10,11,12]. Indeed, the explicit goal of ACT is to enhance psychological flexibility [5,6] by teaching clients to be more open to experiencing private events as they arise (rather than attempting to control them) in leading a life driven by personally chosen values.

Research on psychological inflexibility has typically focused on how individuals respond to aversive private events, but the psychological flexibility model has a broader perspective. Psychological flexibility involves being open to experiencing private events (whatever they be, aversive or otherwise) in the present moment as a conscious human being, persisting or changing in behavior in response to situational demands in pursuit of personally valued directions [13]. It is obvious that individuals will be more open to experiencing appetitive than aversive private events. Yearning to feel good is natural and there is nothing wrong with it. Indeed, leading a valued, meaningful life will conceivably bring about feelings of happiness. However, inflexible attempts at controlling positive cognitions and emotions might become just as problematic.

### 1.2. Experiential Approach: Psychological Inflexibility Regarding Appetitive Private Events

Swails et al. [14] presented the construct of experiential approach as being complementary to the typically studied process of experiential avoidance. Experiential approach can be defined as “involving attempts to contact, sustain, or somehow control positive thoughts, emotions, urges, memories and bodily sensations, as well as the contexts that give rise to them” [14] (p. 528). Experiential approach would be problematic insofar as all of the individual’s resources would be dedicated to it, interfering with values-oriented behavior that, while not pleasant in the short run, might lead to enduring happiness and life satisfaction in the long run [6]. In order to explore this relatively neglected dimension, Swails et al. [14] developed a self-report instrument, the Experiential Approach Scale (EAS). They examined its factorial structure and its relationship to experiential avoidance, and provided evidence of its psychometric properties. Both exploratory and confirmatory factor analyses revealed a two-factor structure with only a modest positive correlation between factors (*r* = 0.24) and no relevant cross-factor loadings. Hence, the EAS was regarded as comprising two separate subscales that would represent different forms of relating to desired affective states.

The first subscale, Anxious Clinging (11 items, Cronbach’s αs ranging from 0.92 to 0.94), would reflect a tendency to capture and hold on to positive emotional states fearing that they disappear. On the other hand, the Experience Prolonging subscale (7 items, Cronbach’s αs ranging from 0.82 to 0.85) refers to simply savoring every joyful moment, however long it lingers. Anxious clinging correlated strongly (*r*s between 0.67 and 0.74) with scores on a widely used measure of experiential avoidance, while Experience Prolonging did so only modestly (*r*s between 0.14 and 0.27). Similarly, convergent validity analyses revealed different patterns of association of each subscale with relevant variables. While the Anxious Clinging subscale correlated positively, at least moderately, with every examined measure of psychological distress and dysfunction (e.g., neuroticism, worry, depression) and negatively with every positively valenced variable (such as subjective happiness and satisfaction with life), the Experience Prolonging subscale showed either insignificant or small positive correlations with the criterion variables. Swails and colleagues concluded that “Anxious Clinging may actively contribute to psychological suffering whereas Experience Prolonging may be inert or perhaps even function as a mild protective factor” [14] (p. 542).

### 1.3. Spanish Adaptation of the EAS

A diversity of measures of psychological inflexibility regarding aversive private events exist [15], with several of them already translated and validated for use with Spanish-speaking populations. However, the EAS is, to our knowledge, the only available instrument specifically devised to measure psychological inflexibility regarding positive cognitions and emotions. The aim of the present study was to translate and adapt the EAS to the Spanish language. The EAS has not yet been adapted to any language other than English, nor its use examined with samples other than those in the original study (US college undergraduates) [14]. In our opinion, having a psychometric measure of experiential approach in Spanish would allow researchers and clinicians to undertake a more comprehensive examination of psychological inflexibility with Spanish-speaking populations. This would contribute to the advancement of cross-cultural research on the underlying psychological processes of the ACT model. In the present study, we conducted a preliminary examination of the EAS factorial structure and convergent validity with a convenience sample of undergraduates from Spain. Specifically, we were interested in determining whether the two-factor structure was maintained, clearly yielding two separate subscales as in the original EAS validation, and whether (and how) these two subscales are associated to experiential avoidance and other criterion variables in a similar fashion as those from the original EAS.

## 2. Materials and Methods

### 2.1. Participants

A convenience sample of 206 college students enrolled in different degree programs at two universities from southern Spain completed a paper-and pencil package of instruments. Inclusion criteria were an age of 18 or older, and being fluent in Spanish. Most participants self-identified as women (79%, *n* = 162; men: 21%, *n* = 43; 1 participant self-identified as gender non-binary). The average age was 20.55 years old (range = 18–37, SD = 5.07). All of them were Spanish citizens and Spanish native speakers.

### 2.2. Instruments

Experiential Approach Scale (EAS [14]). The EAS is an 18-item self-report scale for the assessment of experiential approach. Items are rated on a 7-point Likert-type scale ranging from 1 (Never true) to 7 (Always true). It consists of two subscales: Anxious Clinging (with 11 items, e.g., “When I experience positive emotions, I worry about them fading” and “During my better moments, I expect something will happen and ruin them”), and Experience Prolonging (with the other 7 items, e.g., “If I am in a good mood, I try everything I can to stay that way” and “I do my best to make my good moods last a long time”). Higher scores on the EAS are indicative of a stronger tendency to attempt to control and maintain positive private experiences. The original version of the instrument presents internal consistency values (Cronbach’s α) ranging between 0.92 and 0.94 for Anxious Clinging and between 0.82 and 0.85 for Experience Prolonging. We translated the instrument into Spanish with a parallel back-translation procedure. Items were first translated from English into Spanish by two expert translators, then back-translated into English by another two experts (one of them a native English speaker bilingual in Spanish) and compared with the original ones. Finally, the adequacy of the items to the original construct was assessed by a panel of psychology researchers with experience in ACT and its processes of change. The translated items and their original version in English are included in Table 1.

Acceptance and Action Questionnaire-II (AAQ-II [16]; Spanish adaptation [17]). The AAQ-II is a 7-item self-report questionnaire of experiential avoidance. It measures unwillingness to experience unwanted thoughts and emotions, and inability to behave according to values-directed actions when unwanted cognitions and emotions are present (e.g., “Emotions cause problems in my life’”, “I worry about not being able to control my worries and feelings’”). Items are rated on a 7-point Likert scale (1: never true; 7: always true). Higher scores are indicative of higher experiential avoidance. The Spanish version demonstrated a one-factor structure and good psychometric properties (for the present sample, Cronbach’s α = 0.87).

Positive and Negative Affect Schedule (PANAS [18]; Spanish adaptation [19]). The PANAS is a 20-item self-report scale, divided into two 10-item subscales, one for the assessment of positive affect (PANAS-P) and one for the assessment of negative affect (PANAS-N). Each item presents a word descriptive of either positive (e.g., Active, Proud) or negative (e.g., Irritable, Guilty) affect that participants have to rate on a 5-point Likert scale (1: Not at all; 5: Very much). The Spanish version has good internal consistency data (present sample: Cronbach’s α = 0.84 for both positive and negative affect).

General Health Questionnaire-12 (GHQ-12 [20]; Spanish version [21]). The GHQ-12 is one of the most widely used mental health screening instruments [22]. It comprises 12 items that inquire about the frequency and severity of mental health symptoms and distress, loss of self-confidence, and disfunction in daily activities (e.g., “Have you been feeling unhappy or depressed?”, “Have you been losing confidence in yourself?”, “Have you recently been able to concentrate on whatever you’re doing?”). Each item is rated on a 4-point Likert scale (0-1-2-3). Higher scores in the scale are indicative of higher levels of psychological distress. Previous studies [21] have found that a one-factor solution is adequate. The GHQ-12 showed adequate internal consistency (α = 0.86) with the current sample.

Subjective Happiness Scale (SHS [23]; Spanish version [24]). The SHS is a 4-item scale intended to measure the degree of the participant’s subjective happiness compared to others (e.g., “Compared to most of my peers, I consider myself…”). It is answered on a 7-point Likert scale adjusted to each item (e.g., from “…not a very happy person” to “…a very happy person”), with higher scores indicative of higher levels of subjective happiness. This measure has a one-factor structure and good internal consistency (for the current sample, α = 0.86).

Satisfaction with life scale (SWLS [25]; Spanish version [26]). The SWLS is a 5-item self-report rating scale that assesses global life satisfaction. Items (e.g., “The conditions of my life are excellent”) are rated on a 7-point Likert scale (1: strongly disagree; 7: strongly agree), with higher scores indicative of more life satisfaction. It presents a one-factor structure with adequate internal consistency and temporal reliability. For the sample in this study, Cronbach’s α = 0.80.

### 2.3. Procedure

Before conduction of the study, the Ethics Committee of the University of Jaén approved all of the procedures. Participants were informed of the particulars of the study and asked for voluntary participation during regular classes. Written informed consent was obtained before participation. Participants completed the instrument package in the classroom (paper and pencil format, 15 min approximate duration), and were compensated with course credit according to the ethical guidelines of the university.

### 2.4. Statistical and Psychometric Analysis

Since this is, to our knowledge, the first attempt to adapt the EAS to a different language and cultural context than the one wherein it was first tested, instead of assuming the original factorial structure and testing it through confirmatory analysis, we followed an exploratory factor analysis (EFA) approach with goodness-of-fit indices. Factor analysis was computed with Factor 11.05 [27], while SPSS 20.0 was used for the remaining analyses. Kaiser–Meyer–Olkin (KMO) index and Bartlett’s sphericity test were calculated in order to determine whether data were suitable for factor analysis. Determination of the number of extracted factors was based on optimal implementation of parallel minimum rank factor analysis (MRFA) [28], a procedure involving simulations of 500 datasets by permuting the sample data at random so that numbers of cases and variables are unchanged. Each of these datasets is then subjected to EFA using MRFA, and eigenvalues of the sample are compared to the resulting average eigenvalues for the extracted factors. Retention of factors can be based on sample eigenvalues greater than the average or, preferably, the 95 percentile eigenvalues of the simulated datasets [28]. This procedure has proved to be effective in deciding the number of factors to retain in EFA (outperforming Kaiser’s criteria of eigenvalues > 1 and use of the scree plot). An exploratory robust unweighted least squares factor analysis with a robust Promin rotation based on polychoric correlations (a method adequate for ordinal data, such as those from the EAS 7-point Likert-type scale) was conducted to determine factorial structure and its goodness of fit. The following indicators of goodness of fit were used: RMSEA (root mean square error of approximation), NNFI (non-normed fit index), CFI (comparative fit index), GFI (goodness-of-fit index), as well as their 95% confidence intervals (95% CI). A good model fit was defined as NNFI, CFI, and GFI values > 0.95, and RMSEA value < 0.06 [29,30].

Before conducting statistical analyses, data were examined for missing values. Missing values were 0.4% for all variables, and 0.3% for the EAS. Missing data were imputed with the expectation maximization algorithm in SPSS 20.0, after checking that they were missing completely at random (MCAR) with Little’s MCAR test [31] (χ^2^_(99)_ = 112.688; *p* = 0.164).

The internal consistency of the EAS was explored by the calculation of Cronbach’s α and McDonald’s ω. These coefficients were calculated with Hayes and Coutts’s OMEGA macro for SPSS [32]. In order to explore convergent validity, Pearson correlations were calculated between the EAS and all other measures.

## 3. Results

### 3.1. Factor Structure

The KMO index (0.865) and Bartlett’s sphericity test [*χ*^2^_(153)_ = 2281.50; *p* ≤ 0.001] revealed that the data were suitable for factor analysis. According to the parallel analysis, two factors were retained (see Table 2), each accounting for more variance than the 95 percentile of random percentage of variance.

Table 1 presents the factor loadings for the EAS items according to the robust unweighted least squares factor analysis. The first factor comprised all 11 items from the original Anxious Clinging EAS subscale (items: 1, 2, 3, 5, 7, 8, 13, 14, 15, 16, and 18) plus item 17 from the original Experience Prolonging EAS subscale [14], and accounted for 37.96% of the variance. The second factor comprised all other 6 items from the original Experience Prolonging EAS subscale (items: 4, 6, 9, 10, 11, 12) and accounted for 23.17% of the variance. There were no relevant cross-loadings (no item with factor loadings > 0.32 on both factors). The two-factor solution accounted for a total 61.13% of the variance. Inter-factor correlation was negative and weak (r = −0.094), pointing to two independent EAS subscales, like the original instrument [14], although in the current version, item 17 was allocated to the Anxious Clinging subscale, instead of the Experience Prolonging subscale. This model showed a very good fit, according to the different indicators: RMSEA = 0.0400 (95% CI [0.0170, 0.0401]); NNFI = 0.990 (95% CI [0.987, 0.999]); CFI = 0.992 (95% CI [0.990, 0.999]); GFI = 0.987 (95% CI [0.985, 0.992]).

The first subscale (Anxious Clinging) correlated strongly with the scale total (*r* = 0.877, *p* < 0.001), while the second (Experience Prolonging) did so moderately (*r* = 0.468, *p* < 0.001). Correlation between subscales was insignificant (*r* = −0.014; *p* = 0.846).

### 3.2. Descriptive Data and Internal Consistency

Table 3 presents the descriptive data for each EAS item, as well as for the complete scale and its subscales. For specific items, mean scores ranged from 2.02 (item 18) to 5.78 (item 4), and the standard deviations ranged between 1.24 (item 4) and 1.79 (item 13). Skewness and kurtosis were lower than 1 for the complete scale and for each subscale.

Cronbach’s α for the complete scale was 0.852 (95% CI [0.809, 0.881]), with both subscales showing higher α values. McDonald’s ω for the complete scale was 0.798 (95% CI [0.734, 0.849]), with both subscales showing higher ω values too (see Table 1).

### 3.3. Convergent Validity

Pearson correlations between each of the EAS subscales and all other self-report measures were computed in order to examine convergent validity (see Table 4). Anxious Clinging showed significant strong positive correlations with experiential avoidance (AAQ-II) and mental health symptoms (GHQ-12), and a significant moderate positive correlation with negative affect (PANAS-N). In turn, it correlated negatively and strongly with subjective happiness (SHS), and moderately with life satisfaction (SWLS) and positive affect (PANAS-A). The pattern of correlations was almost the opposite for Experience Prolonging, with positive moderate correlations with measures of happiness, life satisfaction, and positive affect, and a moderate negative correlation with mental health symptoms. Correlations of the Experience Prolonging subscale with experiential avoidance and negative affect were insignificant. In order to determine whether correlation coefficients across EAS subscales differed significantly, we used an updated version of Steiger’s *Z* [33,34] that tests for the statistical significance of differences in dependent correlations. As seen in Table 4, all between-subscale differences in correlation coefficients were statistically significant (all *p*s < 0.001). This pattern of correlations is theoretically coherent and generally consistent with findings from the only previous study employing the EAS [14].

## 4. Discussion

The aim of the present study was to adapt the EAS for use with Spanish-speaking population, and to explore the psychometric properties of this Spanish adaptation with a convenience sample comparable to the one in the original EAS validation study [14].

The Spanish version presented a very similar structure to the original scale, with two independent factors that explained a similar amount of variance as in the original study. These factors were regarded as separate subscales, and showed adequate internal consistency (αs were 0.90 and 0.89 for Anxious Clinging and Experience Prolonging, respectively). There were only small differences between the original EAS and the Spanish adaptation, the most notable being that item 17 (“When I love someone, I can’t get enough of it”) loaded onto Anxious Clinging, instead of onto Experience Prolonging. This might be due to slight differences of nuance in meaning due to translation, with the Spanish version perhaps conveying a romantic tone that is less obvious in the original scale. Nonetheless, other aspects might be at play here, like cultural differences in the expression of affect between Spanish and American populations. In any case, it seems that, for our Spanish sample, the strong emotional attachment suggested in this item fits better with Anxious Clinging. Another difference between both versions concerns the degree of association between subscales. While in the original EAS both subscales were positively, albeit modestly, correlated, in the Spanish version they were not, which adds stronger support to the idea of separate subscales measuring distinct dimensions of experiential approach. This is consistent with the fact the only Anxious Clinging correlated (r = 0.69) with experiential avoidance, unlike the original EAS study (where Experience Prolonging was modestly positively correlated to experiential avoidance as well). Consistent with this, too, is the clearly distinct pattern of correlations found for each subscale in convergent validity analyses. While Anxious Clinging was positively correlated with measures of psychopathology and negative affect, and negatively correlated with all positively valenced measures (positive affect, subjective happiness, and satisfaction with life), Experience Prolonging correlated negatively with psychopathology and positively with all positively valenced measures. These results reveal an even more pronounced differentiation between EAS subscales, with no overlap in their patterns of association with other relevant constructs. Swails et al. [14] suggested that Anxious Clinging and Experience Prolonging might constitute different constructs or facets of experiential approach, each representing a different manner of relating to desired emotions and affective states, and each contributing differently to the development of psychological inflexibility and psychopathology. Our findings suggest that seeking to capture and hold on to positive emotional experiences dreading they will go away (as reflected by Anxious Clinging) would constitute a risk factor for the development and maintenance of negative affect and psychopathology. This way of responding to appetitive private events actually entails the presence of aversive private events (e.g., fear of losing happiness), which somehow places this subscale closer to experiential avoidance. Exploring Anxious Clinging seems necessary in order to gain a more comprehensive understanding of psychological inflexibility and its influence on psychological disorders. Conversely, Experience Prolonging, reflecting efforts to simply prolong or savor joyful moments, would rather work as a protection factor (at least in the non-clinical population). It seems that this way of responding to desired emotional states, being open to fully experiencing them while they last, would entail more positive affect and wellbeing. In any case, more research is needed to confirm these conclusions. It would be interesting to examine the use of the EAS with clinical population to see if the same factor structure is sustained, and whether similar patterns of associations are observed with clinically relevant variables. Likewise, it would be relevant to examine whether the Anxious Clinging subscale, and the EAS more generally, are sensitive in discriminating between clinical and non-clinical population. Based on our findings, we would predict that individuals with clinically significant distress would score higher than non-clinical individuals on Anxious Clinging, and perhaps lower on Experience Prolonging. This, however, is speculative and in need of further investigation.

A growing literature is providing evidence about the paradoxical effects of deliberately pursuing happiness. Research suggests that highly valuing happiness is actually associated with a reduction of positive affect and an increase in negative affect and depressive symptoms [35]. We believe that our findings regarding the two different ways of responding to positive affective states might help to better understand these effects. Extreme and inflexible valuing of happiness (e.g., believing that feeling happy all the time is the normal natural state) may lead to disordered emotion regulation [35]. This pattern of happiness valuing and seeking seems much more like Anxious Clinging than Experience Prolonging. Perhaps the problem is not so much with valuing and seeking happiness, but with how we do it.

Before we conclude, some limitations of the present study are worth mentioning. First, this study examined only one convenience sample of college students from Spain (mostly female). Future studies should examine this version of the EAS with other Spanish samples, including more representative samples of the Spanish population, in order to confirm factor structure and examine metric invariance and other psychometric properties of the scale (e.g., test-retest reliability). Additionally, it remains to be seen whether this Spanish version is applicable with other Spanish-speaking populations from different nationalities. Second, it is also worth noting that this cross-sectional correlational study does not allow for establishing directionality of the observed associations between variables. It may be that higher distress and lower positive affect are the result of a specific way of responding to positive emotions (i.e., by anxiously clinging to them, we make them disappear). It might as well be that different ways of responding to positive emotions result from pre-existing higher levels of distress and lower levels of positive affect (i.e., the less frequent positive emotions are, the more we anxiously cling to them) or it might just be that these different variables are in constant interaction, feeding back to each other. Longitudinal studies would be necessary to shed light on this question. In line with this, it would be relevant to conduct clinical studies to explore the sensitivity of the EAS to psychological interventions aimed at enhancing psychological flexibility, such as acceptance-based interventions.

## 5. Conclusions

The purpose of the present study was to translate and adapt the EAS for use with the Spanish population. The EAS is the first instrument specifically devised to assess a scantly explored aspect of psychological inflexibility: how individuals relate to desired emotions and affective states. To our best knowledge, this is the first attempt to adapt the instrument to a language other than English. This Spanish version shows adequate internal consistency, and replicates the original two-factor structure, with one item (17) loading onto a different factor. This version revealed an absence of inter-factor correlation, and a distinct pattern of subscale correlations with other measures, such that only Anxious Clinging correlated positively with experiential avoidance and measures of distress. These results, generally consistent with the findings in the original validation of the scale, underscore the importance of evaluating Anxious Clinging for a comprehensive assessment of psychological inflexibility. We believe that this Spanish adaptation of the EAS will allow researchers and clinicians to examine this relatively neglected aspect of psychological inflexibility with Spanish-speaking populations. We hope that this will contribute to the advancement of cross-cultural research on the underlying psychological processes of the ACT model.

## Figures and Tables

**Table 1:** Factor loadings from Robust Unweighted Least Squares Factor Analysis, and alpha and omega coefficients.

EAS Item Factor Loadings	Factor 1 (Anxious Clinging)	Factor 2 (Experience Prolonging)
1. Cuando estoy de buen humor me preocupa que algo lo fastidie. [When I’m in a good mood, I worry that something will spoil it]	**0.67**	−0.02
2. Cuando experimento emociones positivas me preocupa que se desvanezcan. [When I experience positive emotions, I worry about them fading]	**0.82**	−0.01
3. La preocupación por perder sentimientos agradables me impide disfrutarlos. [My concern with losing good feelings prevents me from enjoying them.]	**0.80**	−0.10
4. Trato de mantener los sentimientos que disfruto. [I try to hang on to feelings I enjoy]	−0.06	**0.64**
5. Cuando me siento “en la cima del mundo” tengo miedo de dejar escapar dicha sensación. [When I’m feeling on top of the world, I’m afraid to let go of it]	**0.75**	0.21
6. Hago todo lo posible por permanecer feliz todo el tiempo. [I do my best to stay happy all the time]	−0.07	**0.82**
7. Cuando las cosas me van bien pienso que algo malo va a pasar. [When things are going well, I expect something bad to happen]	**0.76**	−0.02
8. Me pregunto por qué mi buen humor es breve. [I wonder why my good moods are fleeting]	**0.73**	−0.00
9. Hago todo lo que está en mi mano por mantener mi buen humor el máximo tiempo posible. [I do my best to make my good moods last a long time]	−0.00	**0.91**
10. Si estoy de buen humor hago todo lo que puedo para mantenerme así. [If I am in a good mood, I try everything I can to stay that way]	−0.02	**0.96**
11. Si pudiera saber por qué estoy feliz, podría hacer que ocurriese más a menudo. [If I could figure out why I am happy, I could make it occur more often]	0.17	**0.56**
12. Cuando me siento bien, intento hacer todo lo que puedo para que dure. [When I’m feeling good, I try to do whatever I can to hang on to it]	−0.02	**0.88**
13. Cuando me importa alguien pienso que lo perderé. [When I care about someone, I think I will lose him or her]	**0.62**	−0.02
14. Desearía poder entender por qué mi felicidad no dura más. [I wish I could understand why my happiness doesn’t stay longer.]	**0.62**	0.12
15. Cuando me estoy divirtiendo siento que la experiencia no durará. [When I am having fun, I feel that the experience will not last]	**0.86**	−0.01
16. Me siento intranquilo cuando me pasan cosas buenas en la vida. [I feel unsettled when good things happen in my life]	**0.63**	−0.04
17. Cuando estoy enamorado nunca tengo bastante. [When I love someone, I can’t get enough of it]	**0.43**	−0.05
18. Durante mis mejores momentos estoy a la espera de que algo ocurra y los arruine. [During my better moments, I expect something will happen and ruin them]	**0.86**	−0.07
Cronbach’s α [95% CI]	0.90 [0.878, 0.921]	0.89 [0.855, 0.911]
McDonald’s ω [95% CI]	0.90 [0.875, 0.921]	0.89 [0.860, 0.917]

**Table 2:** Parallel analysis—minimum rank factor analysis (MRFA) results.

Factor	Real-Data % of Variance	Mean of Random % of Variance	95 Percentile of Random % of Variance
1	39.37	11.55	12.83
2 *	24.24	10.47	11.55
3	6.25	9.65	10.51
4	5.31	8.93	9.62
5	4.06	8.25	8.87
6	3.68	7.64	8.19
7	3.40	7.01	7.56
8	2.90	6.40	6.90
9	2.31	5.78	6.28
10	2.07	5.19	5.72
11	1.60	4.60	5.16
12	1.47	3.99	4.58
13	1.22	3.37	3.99
14	0.98	2.77	3.40
15	0.61	2.13	2.84
16	0.40	1.46	2.13
17	0.14	0.81	1.43

* Advised number of dimensions when the 95 percentile is considered.

**Table 3:** Descriptive statistics for each EAS item, EAS subscale, and total scale scores.

Item	Mean	SD	Kurtosis	Skewness
1	3.31	1.76	−1.06	0.34
2	3.18	1.64	−1.01	0.37
3	2.32	1.44	−0.23	0.85
4	5.78	1.24	3.36	−1.61
5	3.27	1.60	−0.89	0.32
6	4.99	1.57	−0.24	−0.64
7	3.00	1.74	−0.67	0.60
8	2.50	1.63	−0.09	0.96
9	5.15	1.54	−0.06	−0.84
10	5.31	1.49	0.70	−1.02
11	4.75	1.72	−0.90	−0.34
12	5.34	1.47	0.77	−1.06
13	3.01	1.79	−0.78	0.59
14	3.22	1.72	−0.93	0.40
15	2.33	1.43	0.93	1.18
16	2.11	1.48	1.64	1.48
17	2.79	1.65	−0.12	0.86
18	2.02	1.25	1.27	1.34
AC	33.05	13.36	−0.23	0.57
EP	31.31	7.26	0.49	−0.82
EAS	64.37	15.12	0.22	−0.05

Note. AC, Anxious Clinging; EP, Experience Prolonging; EAS, Experiential Approach Scale.

**Table 4:** Pearson correlations between each EAS subscale and other relevant self-report measures.

	Anxious Clinging	Exp. Prolonging	Difference (Steiger’s *Z*)
AAQ-II	0.69 *	−0.06	8.79 *
PANAS—Positive	−0.27 *	0.35 *	−6.42 *
PANAS—Negative	0.38 *	−0.09	4.88 *
GHQ-12	0.62 *	−0.24 *	9.57 *
SHS	−0.56 *	0.32 *	−9.60 *
SWLS	−0.39 *	0.36 *	−7.89 *

Note. AAQ-II = Acceptance and Action Questionnaire-II; PANAS = Positive and Negative Affect Schedule; GHQ = General Health Questionnaire; SHS = Subjective Happiness Scale; and SWLS = Satisfaction With Life Scale. * *p* < 0.001.

## Data Availability

The data presented in this study are available on request from the corresponding author. The data are not publicly available due to privacy reasons.
